# Motivational Reasons for Biased Decisions: The Sunk-Cost Effect’s Instrumental Rationality

**DOI:** 10.3389/fpsyg.2018.00815

**Published:** 2018-05-24

**Authors:** Markus Domeier, Pierre Sachse, Bernd Schäfer

**Affiliations:** Institute of Psychology, University of Innsbruck, Innsbruck, Austria

**Keywords:** need regulation, PSI theory, cognitive bias, irrational decision, sunk-cost effect, decision-making, debiasing

## Abstract

The present study describes the mechanism of need regulation, which accompanies the so-called “biased” decisions. We hypothesized an unconscious urge for psychological need satisfaction as the trigger for cognitive biases. In an experimental study (*N* = 106), participants had the opportunity to win money in a functionality test. In the test, they could either use the solution they had developed (sunk cost) or an alternative solution that offered a higher probability of winning. The selection of the sunk-cost option (SCO) was the most chosen option, supporting the hypothesis of this study. The reason behind the majority of participants choosing the SCO seemed to be the satisfaction of psychological needs, despite a reduced chance of winning money. An intervention, which aimed at triggering self-reflection, had no impact on the decision. The findings of this study contribute to the discussion on the reasons for cognitive biases and their formation in the human mind. Moreover, it discusses the application of the label “irrational” for biased decisions and proposes reasons for instrumental rationality, which exist at an unconscious, need-regulative level.

## Introduction

According to a dolphin, Michael Phelps is a lousy swimmer. According to a cheetah, Usain Bolt runs in slow motion. According to the homo oeconomicus model, people predominantly make decisions irrationally. Do these judgments appear fair to you? In the first two cases, it seems rather clear that an inappropriate frame is being applied to assess these individuals’ ability in swimming or running. However, the third statement still comes up repeatedly when human decision-making abilities are being judged (Magrabi and Bach, 2013). The homo oeconomicus model (Simon, 1955) describes human beings as rational agents, who mostly follow monetary goals, have stable preferences, maximize subjective utility, and ignore sunk-costs.

However, studies show that actual human performance in decision-making deviates from this model (Stanovich and West, 2000), influenced by so-called cognitive biases, departing systematically from the normative standards (Kerr et al., 1996). These biases have an effect on real-life decision-making (for a taxonomy of cognitive biases see Carter et al., 2007; Stanovich et al., 2008). Based on 20 years of past work analyzing 100s of business decisions, Nutt (2002) affirms that 50% of all decisions fail. This number could lead to the impression that the human mind is hopelessly flawed, thereby confirming the accusation of “irrational.”

However, Cohen (1981) has a more positive view of the human reasoning ability. He argues that errors should not be seen as proof of human irrationality. He assumes that errors originate from three areas: the normative system, the interpretation problem, and the external validity problem (Evans, 1993). Errors are rated in relation to a normative system that depends on the understanding of logic and deductive reasoning. However, that system does not necessarily fit to behavior in the real world. The interpretation problem concerns the fact that participants do not necessarily understand the task in the same way the instructor thinks they should. Finally, the external validity problem describes the fact that many tasks in the lab are somewhat artificial and have little in common with real-life tasks. In Cohen’s view, errors can only be rated as such when, under ideal conditions, a person agrees that it is an error. He concludes that no present or future findings in literature or research can lead to the assumption of faulty competence in human reasoning. He instead points to patterns of illusions, which might be active during reasoning and could lead to faulty conclusions.

Evans and Pollard (1981) criticized this line of argumentation, as it offers low practical relevance. Specifically, they consider that realistic tasks could also lead to biases, as personal experiences and emotions can influence the participants’ behavior. Moreover, they express that pointing to illusions, as Cohen (1981) mentions, does not clarify the conditions when an individual is “rational,” or presents a “cognitive illusion,” and what the “ideal conditions” are. Although some of this criticism might apply, Evans (2005) disagrees with the assumption that task designs should be considered artificial, as many well-researched effects in the laboratory have been successfully transferred to the real world.

One approach to deal with the problem of the normative system is distinguishing between two kinds of rationality. The first refers to personal/instrumental reasoning, which is used to achieve one’s goals; the second refers to normative reasoning, which occurs in relation to a normative system (Evans and Over, 1996). These terms have been presented earlier as rationality_1_ and rationality_2_, respectively (Evans, 1993; Evans et al., 1993). This distinction might explain errors, especially when individuals violate the normative rationality and persist with their personal rationality to achieve their goals. Other distinctions of rationality (Stanovich and West, 2000) focus on the difference between evolutionarily developed rationality and individual rationality. These two distinctions might not always correspond, especially in our modern world.

In general, Elqayam and Evans (2011) express doubts about the prevailing role of normativism; this approach assumes that human rationality should be evaluated to the degree that it corresponds to a normative standard. Therefore, they proposed a descriptivist paradigm, which “favors an instrumental, pragmatic conception of rationality, based on achievements of one’s goals” (Elqayam, 2012, p. 628). More specifically, Elqayam (2012) presents a descriptive approach that focuses more on the actual behavior but goes beyond mere description. Descriptivism is based on instrumental rationality, that is, the achievement of personal goals and casts doubt on the supremacy of normative rationality. The latter is not completely excluded but integrated as a subcategory of instrumental rationality. However, if you omit the normative approach, which reference would guide you in the evaluation of behavior? Elqayam (2012) proposes “grounded rationality” to address these concerns. This framework aims to evaluate the rationality of human behavior without referring to a universal standard. Elqayam (2012, p. 43) proposed a first working definition of grounded rationality: “Behavior B is rational for agent A, in epistemic context E, if B facilitates achievement of A’s goals within the constraints of E.” The approach focuses on the achievement of personal goals. The epistemic context includes everything that affects the beliefs and desires of a person, and the constraints cover all physical or mental limitations which human beings might have. Therefore, grounded rationality combines bounded rationality with cognitive variability.

Focusing on the rationality concerning cognitive biases, Tetlock and Mellers (2002) provided another perspective, emphasizing the possibility of reinterpreting cognitive biases and, thus, showing possible “upsides” to these apparently poor decisions. Looking at the example of entrepreneurs demonstrating the overconfidence effect, defined as a systematic overestimation of probabilities (Camerer and Lovallo, 1999), the authors reflect that this bias could also be the reason behind success because it allows them to overcome paralyzing loss aversion, taking more risks in business ventures. This interesting point of view leads to the question of whether biases can involve rationales outside the conventional frame of assessment.

We agree with the position that people sometimes rather follow instrumental rationality (to achieve their goals) than normative rationality. In the present study, we sought to take a closer look at these personal goals and which role they play when people present a “bias” in decision-making, which means that they engage in behaviors that are not expected from the viewpoint of the normative theory. As an example of an effect that is typically referred to as a bias, we chose the sunk-cost effect. Our focus concerning personal goals lies in the role of motivational needs, especially the need for competence. Following the descriptive approach from Elqayam and Evans (2011) and Elqayam (2012), we aimed to determine which behavior is rational for individuals to achieve their personal goals in a given context.

### Understanding the Effects Called “Cognitive Biases”

The term “bias” (as well as “debiasing”) itself implies that there is an undistorted and normative standard from which the behavior deviates (Kerr et al., 1996). Thus, the term in itself is directly judgmental and as it is widely used, shows the dominance of the normative research tradition in the field of decision-making. The term evaluates behavior only from one perspective (normative rationality) and does not sufficiently consider alternative perspectives (i.e., instrumental rationality). Although we do not follow this prejudgment, the term “bias” is used in the following article for two reasons. First, to maintain a connection with the previous research tradition at least in wording (e.g., Heuristics and Biases Program, Tversky and Kahneman, 1974), and second, to clarify that behavior can be “biased” and “useful” at the same time, depending on the perspective.

If we want to find the instrumental rationality in relation to cognitive biases, we need to clearly understand their function, and the purpose they might have. Therefore, the goal should not only be to eliminate or reduce biases but also to understand them and identify their negative and possible positive effects. Despite the fast-growing literature in the field of decision-making, there is still a lack of understanding on how to overcome or how to understand biases (Milkman et al., 2009). According to Fischhoff (1982), the best way to reduce biases would be to have a better understanding of mental processes in general. This would add motivational and emotional aspects to the focus on cognition in decision research. There are interesting developments in this field, for example, the concept of “actively open-minded thinking” (Baron, 1993; Haran et al., 2013). Besides other aspects, it describes a tendency during the decision process to weigh new evidence against a favored belief. As discussed before, if people follow an instrumental rationality when making a decision, they pursue personal goals. Therefore, we need a task design, wherein the variety of personal goals—including motivational aspects—can be observed. Research on the sunk-cost effect, for instance, shows that most of the tasks utilized mainly dealt with cognitive aspects, and therefore, present a low potential for triggering motivational and emotional processes (e.g., van Putten et al., 2010; Hafenbrack et al., 2014). In this regard, after conducting a comprehensive overview of judgment and decision-making research, Weber and Johnson (2009) suggested that it is important to connect more of the research to theories of motivational and emotional processes.

### Psi: A Theory of the Human Mind

To gain a better understanding of the human mind regarding the motivational, emotional, and cognitive processes, a unifying theory is called for. The Psi theory (Dörner, 1999; Dörner et al., 2002; Bach, 2009; Dörner and Güss, 2013) presents a holistic architecture of the human mind. Unlike other architectures, such as State, Operator, and Result (SOAR, Newell, 1987) and the Adaptive Control Thought-Rational theory (ACT-R, Anderson, 1990), the Psi theory integrates the emotional and motivational aspects into the cognitive system. As the theory is particularly extensive, we focused on the concept of motivational needs within the theory.

In the Psi theory, five different needs are distinguished: (1) existential needs (thirst-, hunger-, and pain avoidance), (2) the need for sexuality, (3) the need for affiliation (positive signals from others), (4) the need for certainty (predictability), and (5) the need for competence (active control). Of these five needs, we will focus on the need for competence. Several researchers from various fields view competence as a central need and a drive for human behavior (Bischof, 1987; Adler, 1912; Deci and Ryan, 2000). The need for competence refers to having active control over a situation. This includes the extent to which a person feels capable of handling the problems presented by their environment. This need also encompasses the need for power, control, or autonomy, and is connected to status, self-competence, and self-worth (Dörner et al., 2002). According to Bach (2012), three different kinds of competence can be distinguished: epistemic competence (coping with any specific task), general competence (overall ability to cope with the environment), and effect-related competence (ability to have an effective impact on the environment). Across all three variations, the need for competence is met through the successful satisfaction of other needs, when the person experiences success, especially in demanding situations, and in general when they perceive competence signals. The need for competence is frustrated through failed attempts to satisfy needs in general, the loss of active control, signals arising from failing, and signals of incompetence (Dörner et al., 2002).

Dörner (1999) compares the function of these needs to a reservoir, which has an inherently limited capacity. Positive information fills the reservoir, and negative information leads to a drain. When an individual interacts with the environment, the incoming information is assessed. Depending on the assessment of the situation concerning aspects, such as importance, urgency, threat, subjective competence to cope with the situation, past history of the information, relevance, and anticipation of further development (Detje, 1999), the situation is rated as positive or negative in relation to the level of the reservoir. In the interaction with the environment, the actual level of the reservoir might differ from the target level. If the level drops below the target level, a need becomes active. The further and faster this level drops, the higher the pressure to satisfy the need. If the assessment of the situation is rated as an actual or potential future threat to the need, an urge arises, and the process of need-regulation is initiated. Overall, this process follows a homeostatic principle, aiming at balancing motivational needs in the dynamic environment (Bach, 2009). Satisfaction of the “need pressure” is followed by a pleasure signal; whereas, a high need pressure generates a displeasure signal. Most of these processes occur at the unconscious level. However, the individual can experience the result consciously when it feels “good” or “bad.”

According to the Psi theory, there is a difference between goal-oriented and need-oriented behavior. A goal is defined as a situation associated with a motivational value (Bach, 2009). Of course, every behavior is somehow goal-oriented and motivated in some way. However, the distinction between goals and needs in the Psi theory has another underlying meaning. A goal, in general, describes an entity that directs the behavior. A goal according to the Psi theory is best compared to an “objective goal” (“Sachziel,” Strohschneider, 2003). On the other hand, need-oriented behavior refers to a behavior focused on the satisfaction of needs. For example, a student has to finish a research paper (Dörner and Güss, 2013). The paper represents the objective goal. Completing and submitting the thesis would give the student a strong feeling of competence. However, this goal feels far away when the student is sitting at his/her desk and struggles with the content, therefore receiving a lot of inefficacy-signals. Frustrated, he/she stops working on the paper and starts doing the dishes instead. Washing the dishes does not bring him/her closer to finishing the paper but regulates the need for competence by creating a feeling of being effective in the short run. In the long run, the remaining time to finish the paper runs out. The orientation of the behavior changed from a long-term goal-orientated objective (finishing the paper) to a short-term need-oriented activity (getting efficacy-signals from doing the dishes).

Another approach that also sheds light on the underlying mental processes that occur during the reasoning processes is found in the Meta-Reasoning framework of Ackerman and Thompson (2017). Particularly, the framework refers to the processes that monitor the progress of reasoning and how well a process has unfolded. The states of these processes are experienced as feelings of certainty or uncertainty. The level of certainty experienced is an important aspect of the reasoning process. If a certain level of certainty is reached, a judgment is made. However, the level is not permanently set. According to the duration of the task, the level can sink, thereby leading to judgments with lower requirements for certainty (Diminishing Criterion Model, Ackerman, 2014). During the task, a “feeling of rightness” is experienced if a first solution feels right (Thompson et al., 2011; Ackerman and Thompson, 2017). In the Psi theory, the “feeling of rightness” refers to the level of confidence a person ascribes to a solution option and its anticipated potential for need-satisfaction. This concept is similar to the “good” or “bad” feeling in Psi, which is the result of the need-satisfaction itself. In the Meta-Reasoning framework, certainty plays a central role when implementing an action. The Psi theory also determines the action depending on the need for certainty but additionally, it includes the need for competence and describes action as a result of the interaction between both of these needs (Dörner and Güss, 2013).

### Influence of Motivation on Decision-Making

Decision-making is part of the problem-solving process (Güss and Robinson, 2014). The act of decision-making can be described as the ability to choose one of several alternatives and to act accordingly (Güss, 2004). Mostly, the goal of a decision lies in the future (Hsee and Hastie, 2006). That means, when making a good decision, an individual has to pick an alternative in the present moment, which best fulfills the future requirements of an upcoming situation (Pronin et al., 2008). Güss et al. (2017) emphasize the role of motivation in complex problem-solving; consequently, motivation also has an influence on decision-making.

Decisions do not take place in a vacuum; the needs are influenced by specific characteristics of the situation (Dörner, 1999). Depending on the subjective assessment, the situation is rated as positive or negative with respect to the needs, and therefore, has an influence on the levels in the reservoirs. Subsequently, the individual also looks for aspects in the options, which could mean the fulfillment of their actual needs. Thus, the available options are not only assessed with regard to their goal-reaching potential but also by their need-fulfilling potential. However, this process does not necessarily have to be conscious. Errors can arise when the logic of acting changes from goal-oriented rationality to need-oriented rationality (Dörner, 1996); that is, when the individual unconsciously shifts regarding the decision, moving from the original objective to a sole focus on the satisfaction of one or several needs. The consequence of such a scenario is that an option is selected to satisfy motivational needs, rather than best fulfill the requirements of the situation. This also implies a short-term advantage (i.e., the feeling of competence being maintained) and long-term disadvantage (i.e., the actual goal not being reached). However, there is also the possibility of satisfactorily meeting both needs and present goals with a single decision, especially in cases when the goal equals the fulfillment of needs. As these cases usually do not lead to difficult decisions, because it is a win-win situation, we focused on decisions where the goal and fulfillment of needs differed. We assume that in these cases, regulation can take place over the formation of cognitive biases, as they could serve the (unconscious) preservation of needs or increase the levels in the need reservoirs. Ignoring counterfactual information, over- or underestimating probabilities, and staying with the familiar option are all possible ways to regulate the needs (Dörner et al., 2002; Dörner and Güss, 2013).

### The Sunk-Cost Effect

One of the best-known effects, which is considered a cognitive bias, is the sunk-cost effect. It is defined as a “tendency to continue an endeavor once an investment in money, effort, or time has been made” (Arkes and Blumer, 1985, p. 124). This means that when individuals exhibit sunk-cost effect behavior, they persist with the option which they have already invested in and resist changing to another option that might be more suitable regarding the future requirements of the situation (Arkes and Ayton, 1999; Hastie and Dawes, 2001). Moon (2001) distinguishes two different situations in which the sunk-cost effect occurs. The first is described as “utilization decision,” a decision wherein the decision maker has to choose between two equal alternatives. Roth et al. (2015) illustrated this type of decision with the example of a person who purchased a ticket to a play at the local theater but later gets invited by a good friend to a special Italian dinner. Even when the person prefers attending the dinner, he/she thinks about the sunk cost already paid for the ticket and decides to go to the play.

The second situation is as a “progress decision,” that describes a situation where the decision hinges upon whether or not a chosen pathway should be continued or not. An example of this type of decision is found in the building of the supersonic plane Concorde. Already in the early development stages, the costs rose higher than expected, and the financial success was unclear. Nevertheless, the project was not stopped but further funded because of the amount of money that was already invested (Arkes and Ayton, 1999). Experiences in economics do not help to sustain the sunk-cost effect. A meta-analysis by Roth et al. (2015), which involved solely monetary sunk-cost decisions, revealed that an economic background on the part of the participants does not have a significant influence on utilization decisions.

There are different approaches to explain the occurrence of the sunk-cost effect. van Putten et al. (2010) differentiated between individuals with a “state orientation,” who struggle to let go of past events, and individuals with an “action orientation,” who seem relatively untroubled by past events. They found that state-oriented decision-makers were more prone to exhibit the sunk-cost effect. Other researchers bring in further aspects, such as people not wanting to appear wasteful (Arkes and Blumer, 1985), the effect of mental accounting (Thaler, 1985), or the escalation of commitment (Schaubroeck and Davis, 1994; Carter et al., 2007). Most of the time, sunk-cost effect studies involve hypothetical scenarios. In some cases, a situation is described which places the participants in a position where they have to make a decision (“Imagine you are the CEO of …”) (see van Putten et al., 2010; Hafenbrack et al., 2014) concerning whether a course of action or project they have already invested in should be continued and, therefore, supported financially, even when a competitor presents a more promising solution. In these cases, participants often have to make a decision involving millions of theoretical dollars of investment. However, most of these cases seem rather artificial, as ‘normal’ participants suddenly are required to imagine that they are a CEO with great responsibility. Moreover, most of these cases only address money (Roth et al., 2015), and focus less, if at all, on time or effort. Even when they do, it is only within a hypothetical frame.

### Self-Reflection as a “Debiasing” Intervention

There are various approaches that could improve decision-making, preventing decision-makers from committing the so-called cognitive biases (Soll et al., 2015). These approaches can be summed up as “debiasing” interventions. As discussed before, the problem with the term “bias” also concerns the term “debiasing” which is used to describe interventions that aim to eliminate biases. This process should eventually lead to a decision outcome which corresponds to a normative rationality. However, despite the focus on normative rationality, biases can also make the decision-maker aware of the different rationalities he or she might follow in a given situation.

One of these debiasing interventions is to initiate a process of self-reflection to unveil biases running at the unconscious level (Donovan et al., 2015; Phillips et al., 2016). Hafenbrack et al. (2014) found that including a meditation-based intervention to prevent the sunk-cost effect seems to be an effective approach to lead participants to focus more on the actual situation, and less on the past, where the sunk costs took place. Another approach to initiate a process of self-reflection is to let decision-makers assess the situation from an external point of view. There is a difference between making decisions for ourselves and for others. Various studies on the concept of self-other decision-making have shown that individuals making a decision for others focus on fewer attributes and make the decision more readily (Kray and Gonzalez, 1999), focus less on feelings (Hsee and Weber, 1997; Hong and Chang, 2015), and make more risk-averse decisions when risk-aversion is pertinent to the situation (Stone et al., 2013). A debiasing intervention designed by Kahneman et al. (2011) aims at exhibiting this effect, as the individual is forced to evaluate the decision from an external point of view (“How would a new CEO decide?”). In the case of the sunk-cost effect, this should disclose unrelated motivational factors, as the decision-maker has to analyze the decision from an external point of view. In our study, the aim of the debiasing intervention was to make participants aware of the normative rationality they follow when deciding for others and to adopt this normative rationality when deciding for themselves. Therefore, this change of perspective should draw attention to aspects that are important for reaching the objective goals of a situation, and less on subjective aspects which are only important from the view of the decision-maker. Thus, the intervention should ultimately lead to a more goal-oriented decision (Strohschneider, 2003). As a general point, Strough et al. (2016) highlight the importance of understanding the mental models and existing beliefs of participants when designing an intervention.

### Aims of the Present Study

The current theoretical background suggests that there is still little understanding of how and why these effects, in our example the sunk-cost effect, occur. However, a better understanding would have significant positive effects, leading to better decision-making (up to 7% higher return after reducing the effect of biases in business decisions, Kahneman et al., 2011). Therefore, the present study aims to address the following questions: which goals do people follow when they demonstrate instrumental rationality (Evans and Over, 1996), and what role does the unconscious need regulation play in the formation of these goals? What would a structured experimental design that aims to capture the complexity of a real-life situation look like? (Dörner and Funke, 2017). Finally, can an intervention influence the rationalities that the decision-maker follows? We conducted a study designed to analyze participants’ decisions in a more realistic sunk-cost scenario. Participants were asked to choose between a sunk-cost option (SCO) and an alternative option (AO), whereby one group received an intervention with the aim of interrupting the choice of the SCO.

We derived the following hypotheses: biased decisions (i.e., taking the SCO) have their root in unconscious need regulation, triggered by sunk costs. As proposed in the Psi theory (Dörner, 1999; Bach, 2009; Dörner and Güss, 2013), we assumed that need regulation is positively linked to choosing the SCO (Hypothesis 1). Accordingly, we hypothesized that participants who choose the SCO show lower levels of self-reflection (Hypothesis 2a), achievement motives, and self-control (Hypothesis 2b). Research indicates that biases can be prevented by inducing a process of self-reflection (Hsee and Weber, 1997; Kahneman et al., 2011; Hong and Chang, 2015). Consequently, we hypothesized that an intervention fostering self-reflection prevents decision-makers from choosing the SCO (Hypothesis 3).

## Materials and Methods

To motivate the participants, we created a more “involving” experimental scenario to test the hypothesized mechanism, in which all three aspects of the sunk-cost effect were present. Participants were required to work on their own project (effort), into which they invested their own time and money, and had to decide whether to persist with their own project or choose an alternative and more promising option, in an attempt to win the remaining sum of their investment. Following the understanding of Greitemeyer et al. (2005), when participants chose their own option, in which they had already invested, even when the AO offered a higher chance of winning money, their choice was rated as a sunk-cost effect.

Against the background of the Psi theory concerning psychological needs, we aimed to unveil the underlying mechanisms and reasons why people choose the SCO. The question was: can we observe need-regulation through the actions of participants who choose the SCO (SCO-Selectors) reporting greater need-satisfaction than participants who choose the Alternative Option (AO-Selectors)? Moreover, we tested an intervention (Kahneman et al., 2011) designed to induce a change of perspective regarding the decision and reduce the sunk-cost effect, with the aim of generating a more goal-oriented, and less need-oriented, decision.

### Participants

From a total of 138 adult participants who were recruited for the experiment, 13 were excluded due to missing data (*n* = 2), unfinished construction (*n* = 4), misunderstanding of the task, error in the experimental flow (*n* = 3), and knowledge of the task (*n* = 4). Another 19 participants from the pre-testing phase were also excluded, as the task was slightly adapted. The remaining 106 participants (40 men and 66 women; mean age = 21.75 years; *SD* = 3.0; range = 18–37 years, 95% psychology students) were included in the analysis. Participants were recruited from the campus of the University of Innsbruck.

### Materials

Participants completed the Self-Control Scale (SCS-K-D) (α = 0.82) from Bertrams and Dickhäuser (2009) (13 items). They also completed the Self-Reflection and Insight Scale (SRIS) (Grant et al., 2002), which is divided into the Self-Reflection Scale (α = 0.85) (12 items) and the Insight Scale (α = 0.84) (eight items). The Achievement Motives Scale (Lang and Fries, 2006), (10 items), which is divided into two dimensions, hope of success (five items, α = 0.77) and fear of failure (five items, α = 0.81), was used to identify the motives of participants. The Construction Task was adapted from the classical sunk-cost cases provided by Arkes and Blumer (1985) and van Putten et al. (2010). In the intervention, participants had to decide for a fictional ‘other person’ to use the construction that the participant has made or the alternative construction (“Please put yourself in the role of a person who has not worked on your construction and has to make the same decision as you. In your opinion, which construction should the person take?”). The intervention and the actual decision were binary-coded (adopting the self-built construction versus taking the alternative construction).

The assessment of Competence Satisfaction (α = 0.90, seven Items) was adapted from Bach (2009) and Dörner and Güss (2013), and the estimated probability of the success of the construction was rated on a number scale ranging from 0 to 100% (where 0% equaled no chance of winning the money with the construction, and 100% equaled a safe win).

The items were preceded by the sentences: “With my solution/the alternative solution, I associate…” (...the feeling of being in control of this situation;... the feeling of being effective in my actions;... a positive feeling, because I think that I’m successful with it;... generally a positive feeling); and “My solution/the alternative solution …” (...gives my self-esteem a positive feeling;...gives me the feeling of being able to do something actively;...makes me feel self-determined). Items were scored on a seven-point Likert scale ranging from 1 (strongly disagree) to 7 (strongly agree).

The alternative construction was coded with a 50% chance of winning. Pre-tests showed a 30% chance of winning when the participants’ own constructions were used. The functionality of the constructions was physically tested at the end of the experiment.

### Procedures

The researcher welcomed the participants and led them to their seats. A maximum of three participants was tested at the same time. The working spaces were divided by partitions to prevent any communication. After a short introduction, in which participants were asked not to use their phones or talk to each other, they were required to wear earplugs. Afterward, participants were randomly assigned to the group with or without the debiasing intervention.

In both conditions, participants completed a demographic survey, the SRIS from Grant et al. (2002), the SCS-K-D from Bertrams and Dickhäuser (2009), and the Achievement Motives Scale from Lang and Fries (2006). Then, participants were assigned a decision-making task, adapted from the cases of Arkes and Blumer (1985) and van Putten et al. (2010). In this case, participants were asked to take the role of the project leader in a company that produced packing solutions. The goal was to design and build a construction, within 30 min, which could protect a raw egg when dropped from a height of 3 m. For this task, participants were provided with 15€ and presented with a selection of different materials that they could purchase [a straw (0.50€), 0.5 m of rope (2€), 4 cm of tape (0.50€), one napkin (0.50€), a plastic cup with a volume of 0.2 l (2.50€), a DinA4 paper (1€), a balloon (2€), a plastic bag (2.50€), a cotton pad (0.50€)]. They were allocated 10 min to think about their construction and buy the materials they would need. There was no restriction on how many units of an item they could purchase. The incentive to build economic solutions was that participants could keep any funds remaining in their budget after purchasing the materials but only if their solution worked (i.e., if the egg survived the 3 m drop). For example, with an investment of 10€ in materials, they could earn 5€ if the egg survived the drop. Subsequently, participants’ understanding of the conditions of the task, and the possibility of winning money was checked using a short survey.

After the 10-min planning and purchasing phase, the 20-min construction phase began. In this phase, participants once again had an opportunity to order new materials. At the end of the construction phase, participants were notified that there was another type of construction available, which they could use for the drop. They could not view the construction but were informed that this alternative construction would perform better than the average self-created construction. They then had to weigh up the possibility of success of their own construction set against this other alternative.

The control group had to decide which of the two constructions they would like to use for the drop: their own, on which they had planned and worked for 30 min and had invested money, or the alternative construction. The experimental group, however, received a short intervention (adapted from Kahneman et al., 2011) before their choice. They had to consider what a new project leader, who had not worked on either of the solutions, should choose. They noted down the reasons for their decision regarding this extra choice. After making this decision, the participants of the experimental group then had to make the choice for themselves.

Subsequently, both groups assessed the two solutions for their Competence Satisfaction. After assessing the constructions and choosing between them, the functionality test was conducted. If participants chose the AO, a generator with a 50% chance of winning was used to decide if they could keep the rest of their budget. If participants chose their own construction, the researcher dropped their construction from a height of 3 m. The remaining budget was given to them only if the egg survived the drop.

### Design

Participants were assigned to conditions in a 2 × 2 design (experimental group/control group × sunk-cost option selectors/alternative option selectors). The experiment was designed to test whether an intervention inducing a self-reflection process leads to a reduced selection of the SCO in comparison to the control group without an intervention. Moreover, after the choice was made, the reasons for the decision were assessed on a need-regulative level. Responses to scales for self-control, self-reflection, and achievement motivation were obtained to check for differences between the groups.

## Results

**FIGURE 1:**
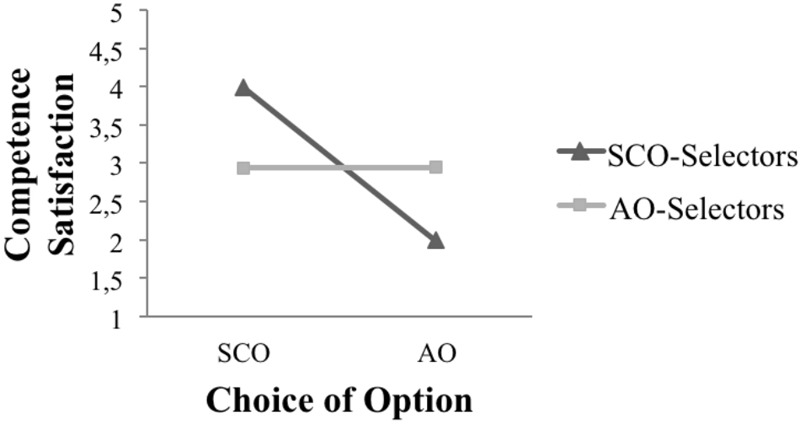
Rating of competence satisfaction for the two options.

To check Hypothesis 1, which assumed that need regulation is positively linked to choosing the SCO, the assessment of Competence Satisfaction was analyzed (**Figure 1**). Interestingly, the SCO-Selectors assessed the SCO as more satisfactory in terms of competence (median = 4.00) than did the AO-Selectors (median = 2.86, *U* = 201.5, *p* < 0.001). According to Cohen (1992), this indicates a medium effect (*r* = 0.40). A significant difference was also found in the assessment of the AO. The AO-Selectors rated the AO higher (median = 2.86) than did the SCO-Selectors (median = 1.86, *U* = 244.5, *p* < 0.001). According to Cohen (1992), this indicates a medium effect (*r* = 0.36). This difference was not only found between the two groups but also within the group of SCO-Selectors, who rated the SCO significantly higher (*M* = 4.01, *SD* = 0.86) than the AO (*M* = 2.07, *SD* = 0.90, *t* = 15.46, *p* < 0.001, *n* = 92, *r* = 0.85). No such difference was found in the assessment of the AO-Selectors. Running a Wilcoxon signed-rank test, no significant differences were found between the ratings of the SCO (median = 2.94) and those of the AO (median = 2.95, *z* = -0.189, *p* = 0.850, *n* = 14, *r* = 0.05).

**FIGURE 2:**
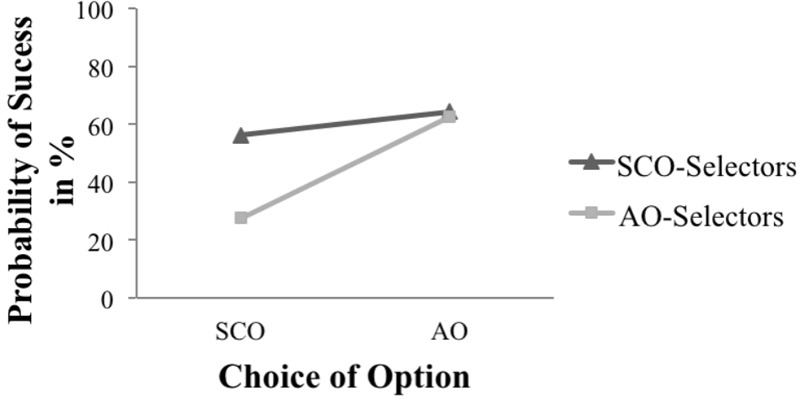
Subjective probability of success for the two options.

Concerning the success probability of the option, which was assessed on a percentage scale (where 0% meant no chance of protecting the egg, and 100% equaled an effective, safe construction), in both groups, participants assessed the AO as significantly safer (*M* = 64.06, *SD* = 15.37) than the SCO (*M* = 52.38, *SD* = 21.90, *t* = -6.42, *p* < 0.001, *N* = 106, *r* = 0.53). This effect could also be seen when the success probability was analyzed depending on the decision (see **Figure 2**). Following expectations, a Wilcoxon signed-ranks test indicated that the AO-Selectors rated the AO significantly higher (median = 61.00) than the SCO (median = 26.00, *z* = -3.30, *p* < 0.001, *n* = 14). According to Cohen’s effect size (1992), *r* = 0.88 indicates a strong effect. Surprisingly, the SCO-Selectors also rated the AO significantly higher (*M* = 64.27, *SD* = 15.88) than their own chosen SCO (*M* = 56.13, *SD* = 20.67, *t* = -4.68, *p* < 0.001, *n* = 91, *r* = 0.19).

The SCO-Selectors assessed the SCO with a higher success probability (median = 59.50) than did the AO-Selectors (median = 26.00, *U* = 161.5, *p* < 0.001). According to Cohen (1992), this indicates a medium effect (*r* = 0.44).

There were no significant differences between the experimental and control groups with respect to the scores on the SRIS [Levene-test: *F*(1,104) = 1.417, *p* = 0.237, *N* = 106] (Hypothesis 2a). The Self-Control Scale [*t*(104) = 0.107, *p* = 0.915] and the Achievement Motives Scale for the dimensions ‘hope of success’ [*t*(104) = 1.594, *p* = 0.114] and ‘fear’ [*t*(104) = 0.527, *p* = 0.599] also showed no differences between the groups (Hypothesis 2b). The amount of investment in the construction and the decision revealed no significant findings (χ^2^ = 0.354, *p* = 0.552, *df* = 1).

Moreover, the analysis did not reveal any significant differences between the SCO-Selectors and the AO-Selectors regarding the SRIS (*U* = 461.0, *p* = 0.087, *r* = 0.17), the Self-Control Scale (*U* = 595.5, *p* = 0.651, *r* = 0.04), or the Achievement Motives Scale in the dimensions ‘hope of success’ (*U* = 556.5, *p* = 0.410, *r* = 0.08) and ‘fear’ (*U* = 441.5, *p* = 0.058, *r* = 0.18).

To check for the effect of the intervention (Hypothesis 3), we took a closer look at participants in the experimental group. Before deciding on whether to choose their own option (SCO) or the AO, they had to decide on behalf of an external person, who was not involved in the process. The majority chose the AO (86.5%, *n* = 45), rather than the SCO (13.5%, *n* = 7, exact binominal test, two-sided, *p* < 0.001, *n* = 52).

However, when making the actual decision (which option to use for the egg test), the groups showed similar results. In the experimental group, 45 participants chose the SCO (86.5%) and only seven, the AO (13.5%). Running a two-sided binomial test revealed a significant deviation from the expected 50% distribution (*p* < 0.001, *n* = 52). In the control group, a similar picture was observed: 47 participants chose the SCO (87%) and seven, the AO (13%). A two-sided binominal test also showed a significant deviation from the expected 50% distribution (*p* < 0.001, *n* = 54). A binominal test was conducted taking 87% of the control group as a reference point; the analysis did not show any significant difference between the two groups (*p* = 0.522, *n* = 52).

Taken together, the results show that 33.7% of the 92 SCO-Selectors were successful and won an average of 4.76€ (median = 5.00); 71.4% of the 14 AO-Selectors were successful and won an average of 4.05€ (median = 4.00). The difference was not significant (*U* = 107.00, *p* = 0.136).

## Discussion

In the present study, we placed participants in a situation where they were confronted with two options; one of these was the so-called SCO (Arkes and Blumer, 1985; Greitemeyer et al., 2005). With the materials that could be purchased from a given budget, the aim was to build a construction to protect a raw egg from a 3-m drop. They could only “win” any money left over from the budget if the egg survived the drop without damage. After building their own construction for the drop-test, participants were confronted with an AO, with a reportedly higher possibility of securing the remaining money. Subsequently, they had to decide whether they wanted to use their own construction or the alternative construction for the drop. With either option, participants had the chance to win the remaining money from the budget. If the participants persisted with their own option, on which they had invested time, money, and effort, despite being informed that this would generate, on an average, a lower chance of winning, it was rated a “sunk-cost effect.”

To take a deeper look into the reasons behind the decisions made, we analyzed the choice from a psychological needs perspective, based on the Psi theory (Dörner et al., 2002; Bach, 2009). The focus of this analysis was on the need for competence. The need for competence describes one’s perception of active control over the environment. Signals of effectiveness add a positive value in terms of competence; signals of ineffectiveness do the opposite, decreasing the levels in the competence “reservoir” (Dörner and Güss, 2013). Having invested in vain in a particular option would send a significant signal of ineffectiveness to the participant. Therefore, choosing the SCO would protect the individual from this negative feeling, in the short term (Dörner, 1996). Taking this into account, we analyzed if need-regulation can better explain why people make economically “irrational” decisions (Simon, 1955), assuming a mechanism of need-regulation, which leads to biased decisions, from the normative point of view (Dörner, 1996, 1999; Strohschneider, 2003; Dörner and Güss, 2013). From this perspective, the decision-maker favors the option which adds most value concerning their psychological needs, rather than the option which is likely to maximize their chances of winning. From the viewpoint of rationality, participants followed an instrumental rationality when choosing an option.

The most remarkable result was that when participants had to make the decision, it did not matter which option was more promising regarding likely returns. This directly violates the expectation that an individual will act according to the homo oeconomicus model (Simon, 1955). Participants rather chose the option which potentially maintained or boosted their feeling of competence. Results from the list of reasons for their choice indicated that the SCO-Selectors made their choice to check their effectiveness in building the construction. However, the AO-Selectors lacked such confidence in their construction. When they expected that their construction would fail to protect the egg, using the SCO equaled a possible threat to their feelings of competence. The data showed that in this case, participants chose the AO because this option gave them a higher chance of winning while also avoiding the potential signal of ineffectiveness from the failure of their own construction.

The intervention that aimed at changing participant’s rationality by inducing self-reflection (Donovan et al., 2015) worked in the first stage (Kahneman et al., 2011); participants reported that the other person should take the AO, given it was likely the “better” one. In the second stage, however, when they had to choose for themselves, this prior normative rational insight did not prevent them from choosing their own, reportedly less successful, option. According to Evans and Over (1996), participants showed more normative rationality (acting according to the homo oeconomicus model, selecting the option with the maximum chance of winning) when choosing for others but switched to an instrumental rationality (achieving personal goals) when choosing for themselves. As participants reported a higher need satisfaction from their chosen option, we suppose that regulation of the need for competence might be an important aspect concerning the formation of personal goals.

### Limitations and Outlook

The intervention showed no impact on preventing participants from choosing the SCO. Even when making the normative rational choice for someone else, they still picked the SCO when they made the decision for themselves. The reason could be that the intervention did not involve the satisfaction of needs. Even when participants were able to reflect on the situation consciously, they still had an (unconscious) urge to regulate their needs. An intervention designed to satisfy needs before the decision is made regarding their own choice might lead to better results.

As proportionally so many participants chose their own option, we had to use some non-parametric tests. Our explanation for this imbalance lies in the task itself. In artificial cases, more people choose the rational option (for instance, between 29 and 44% resisted the sunk-cost bias in the control groups of Hafenbrack et al., 2014). However, in our more “realistic” scenario, persisting with their own SCO seemed to have a stronger pull. It would be very interesting for future research to examine whether there is a financial threshold where participants would be more influenced by the option with a higher possibility of winning than the regulation of needs. Future research could conduct a systematic research on various cognitive biases and the need-regulative function during the decision process.

The presented findings are correlational and are based on self-report measures. Therefore, it remains unclear if need-regulation leads to the sunk-cost effect, the sunk-cost effect triggers the need-regulation, or both. Future research should include these considerations.

## Conclusion

Is the observed behavior irrational? The answer depends on the point of view; from a normative view, some participants acted irrationally when they did not choose the option which maximized their chance of winning. However, when taking the instrumental rationality (Evans and Over, 1996) into account, there could be a different interpretation. Instrumental rationality states that a person acts to achieve his or her goals. In the given situation, participants worked on their solution, invested time, money, and effort. Being confronted with the thought of having done this in vain might be a strong negative signal to their competence. Subsequently, along with the goal to choose the option with the highest chance of winning, the goal to regulate the need for competence also arises. Therefore, choosing their own option, even when it had a lower chance of winning the money, might serve a personal goal: to maintain or even increase their feeling of competence. Is the sunk-cost effect a bias? Yes and no. From a normative point of view, involving sunk costs into a decision is a deviation from the normative model, and therefore, a “bias.” However, this only applies from the normative perspective. From the viewpoint of instrumental rationality, the sunk-cost effect serves personal goals which can differ from the normative standards. Therefore, reflecting back on the framework of grounded rationality (Elqayam, 2012), a “bias” can be seen as a behavior that is rational to the decision maker in an epistemic context, if the “bias” facilitates the achievement of the decision maker’s goals within his or her physical or mental limitations.

According to Strohschneider (2003), we can observe a shift from a goal-oriented behavior to a need-regulation oriented behavior. Typically, this shift should not pose a problem but when the decision-maker is not aware of it, he or she might gain a short-term regulation of the need but fails to maximize their chances of attaining their objective in the long run. Additionally, the goals arising from the need-regulation do not necessarily have to be conscious to the decision-maker. Therefore, the final question would be: even when the exhibited behavior is in some way rational, is it the way I want to or should act in the given situation?

## Ethics Statement

This study was carried out in accordance with the recommendations of “The Board for Ethical Questions in Science of the University of Innsbruck” with written informed consent from all participants. All participants gave written informed consent in accordance with the Declaration of Helsinki. The protocol was approved by the “The Board for Ethical Questions in Science of the University of Innsbruck.”

## Author Contributions

MD and BS conducted the data collection, data analysis, and the writing of the manuscript. All authors were substantially involved in the planning of the study, the interpretation of data, and revision of the article.

## Conflict of Interest Statement

The authors declare that the research was conducted in the absence of any commercial or financial relationships that could be construed as a potential conflict of interest.
